# Absolute Primary Nanothermometry Using Individual Stark Sublevels of Rare-Earth-Doped Crystals

**DOI:** 10.1021/jacs.6c17449

**Published:** 2026-09-27

**Authors:** Allison R. Pessoa, Thomas Possmayer, Jefferson A. O. Galindo, Luiz Fernando dos Santos, Rogéria R. Gonçalves, Leonardo de S. Menezes, Anderson M. Amaral

**Affiliations:** † Department of Physics, 28116 Universidade Federal de Pernambuco, Recife, Pernambuco 50740-540, Brazil; ‡ Federal Institute of Education, Science and Technology of Pernambuco, Recife, Pernambuco 50740-545, Brazil; § Chair in Hybrid Nanosystems, Faculty of Physics, Ludwig-Maximilians-Universität München, München 80539, Germany; ∥ Department of Chemistry, Center of Nanotechnology and Tissue Engineering- Mater Lumen Laboratory, Faculty of Philosophy, Science and Letters of Ribeirão Preto, 28133 University of São Paulo, Ribeirão Preto, São Paulo 14040-901, Brazil

## Abstract

We present two independent optical methods for absolute primary thermometry using rare-earth-doped nanoparticles. Both approaches rely exclusively on the internal energy levels and population dynamics of the dopant ions, eliminating the need for external temperature references. We experimentally demonstrate the concepts by using Y_2_O_3_: Yb^3+^/Er^3+^ nanoparticles, exploiting Boltzmann distribution between individual Stark sublevels of the Er^3+^ ions, emitting in the green spectral region (∼550 nm) and in the near-infrared spectral region (∼1600 nm). Our strategy establishes rare-earth-based luminescence thermometers as genuine absolute primary probes, conceptually comparable to Johnson noise and acoustic gas thermometers, but with the fundamental advantage of possibly being employed at the nanoscale, potentially down to the single-ion limit, with optical readout and over wide temperature ranges.

## Graphical abstract

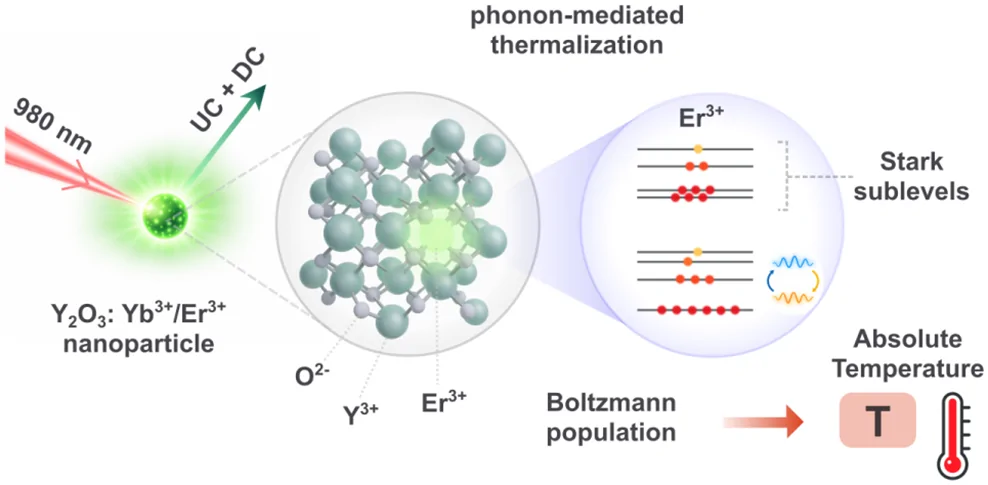

## Introduction

Temperature is a fundamental physical quantity that governs phenomena such as chemical reactivity, phase transitions, and biological processes. Thermometers that rely on a well-defined physical law (equation of state) relating the thermometric parameter (*R*) to the thermodynamic temperature (*T*) are termed as *primary thermometers*.^1^ In this case, the relationship between *T* and *R* is ideally independent of unknown temperature-dependent variables. With the Kelvin scale redefinition in 2019,^1^ the Bureau International des Poids et Mesures (BIPM) introduced the terminology “absolute primary thermometry” for measurement techniques that determine *T* without reference to any external temperature, and “relative primary thermometry” for techniques that require one or more fixed temperature points to establish key parameters of the equation of state. Standard examples of absolute primary thermometers include acoustic gas thermometers (AGT),^2^ Johnson noise thermometers (JNT)^3^ and blackbody radiation-based thermometers.^4^ In particular, AGTs are currently used for the state-of-the-art determination of the triple point of water, achieving relative uncertainties on the order of 10^–6^.^2^ More recently, Brillouin scattering by gases in hollow-core optical fibers has been also proposed for absolute primary approaches,^5^ as well as Stokes and anti-Stokes shifts in single quantum dots.^6^

At the nanoscale, however, accurate temperature measurements pose a significant challenge due to the limited spatial resolution of conventional thermometers and the invasive nature of most contact-based techniques.^7^ As a result, there has been a growing interest in the development of luminescent nanothermometers that can convert temperature changes into optical signals in a minimally invasive fashion.^8^ Among various luminescent nanothermometric platforms, such as quantum dots,^9^ organic dyes^10^ and single nanodiamonds containing color centers,^11^ rare-earth-doped nanoparticles have emerged as particularly attractive candidates.^12^ These systems benefit from the unique optical properties of trivalent lanthanide ions (Ln^3+^), including sharp emission lines, long excited-state lifetimes, and high photostability.^13,14^ Thermometry using Ln^3+^-doped particles has been demonstrated across a wide temperature range, spanning from cryogenic temperatures (>2.5 K)^15–19^ to temperatures exceeding 1000 K.^18–21^ In particular, Yb^3+^/Er^3+^-codoped crystalline solids are widely used for relative primary thermometry around room temperature,^22^ for which most studies rely on the Er^3+^ thermally coupled spin–orbit manifolds ^2^H_11/2_ and ^4^S_3/2_, emitting in the green spectral region.^23^ More recently, Possmayer et al.^17^ exploited the Stark sublevel structure of the ^4^S_3/2_ manifold for relative primary thermometry in the cryogenic range. Similarly, Boldyrev et al.^16^ investigated the Stark structure of the ^4^I_13/2_ manifold emitting in the near-infrared range (NIR) also for cryothermometry.

Despite their advantages, most current implementations of Ln^3+^-based luminescent nanothermometers are limited to relative primary methods.^23–25^ In practice, this means that the thermometric parameter must be calibrated against one or more temperature points, determined by external probes. This approach assumes that the calibration remains valid under possibly varying experimental conditions. However, several factors are known to influence the calibration parameters, both in ensemble experiments^26^ and, in particular, at the single-nanoparticle level. The latter includes effects of the surrounding medium,^27,28^ photonic effects in the host medium,^29,30^ and the presence of power-dependent intruding luminescence bands,^31,32^ all of which may compromise the reliability of such approaches.

Toward the realization of absolute primary thermometers working at the nanoscale and over a wide temperature range, the work of Souza et al.^33^ represents an important step using Eu^3+^-doped systems. The authors exploit the Boltzmann population distribution among closely spaced spin–orbit levels near the Eu^3+^ ground state. By tuning the excitation wavelength from 570 to 640 nm, they selectively addressed the initial manifold ⟨*i*| involved in the optical transition ⟨*i*| → ^5^ *D*_0_. The thermodynamic temperature was then determined from the emission spectra of the ^5^ *D*_0_ → ^7^ *F*_4_ transition after the controlled excitation. Because the method depends on this specific arrangement of energy levels, extending it to other Ln^3+^ ions is not straightforward. Here, we propose two alternative procedures for realizing absolute primary optical nanothermometry that rely exclusively on the emission spectrum, requiring no variation in the excitation wavelength. We demonstrate both approaches by exploiting the Stark sublevel structure of Er^3+^ ions in Y_2_O_3_: Yb^3+^/Er^3+^ nanoparticles under 980 nm illumination, thoroughly analyzing their respective advantages and limitations.

## Results

### Proposed Methods for Absolute Primary Thermometry Using Stark Sublevels

When Ln^3+^ ions are doped into crystalline hosts, their degenerate spin–orbit manifolds are split into closely spaced Stark sublevels, typically separated by energies on the order of 10^2^ cm^–1^.^34^ Upon electronic excitation to a given manifold, electrons rapidly thermalize among its Stark sublevels (and possibly with adjacent manifolds) on the time scale of picoseconds to nanoseconds, which is usually much shorter than the characteristics radiative lifetimes.^35,36^ The thermally accessible levels within a given temperature range are said to be Thermally Coupled (TC). Radiative transitions from the Stark sublevels of the excited manifold to sublevels of lower-lying manifolds give rise to luminescence bands composed of many Stark–Stark spectral lines. Under thermal equilibrium conditions, Boltzmann distribution dictates that the luminescence intensity ratio (LIR) between two TC Stark spectral lines follows a single exponential function^36^

1 $$R_{\mathrm{Stark}}\left(T\right)=C\cdot \exp\left(-\frac{\Delta E}{k_{B}T}\right)$$

where *C* is a constant that depends on the radiative decay rates from the two emitting TC sublevels and Δ*E* is their energy difference.

To realize an absolute primary thermometer based on the Boltzmann distribution among the Stark sublevels, it is necessary to know Δ*E* and *C* from eq 1 independently of external thermometers. By analyzing the peak position of the Stark lines in the luminescence spectrum, we can confidently determine Δ*E* with uncertainties on the order of 0.5 cm^–1^ or less,^17^ limited by the host matrix and/or by the resolution of the spectrometer used for recording the spectra. On the other hand, when measuring the spectrum with photon-counting devices, as with CCD cameras, the parameter *C* can be written as *C* = *g*_2_ *A*_20_/*g*_1_ *A*_10_, where *g*_2_(*g*_1_) is the degeneracy of the higher­(lower)-lying TC sublevel and *A*_20_(*A*_10_) is the radiative transition rate of the corresponding Stark–Stark transition.^36,37^ Calculating these rates from first-principles is difficult for several reasons. For instance, electric dipole transitions are parity-forbidden between 4*f* orbitals, such that the radiative emission becomes allowed through crystal-field–induced mixing with opposite-parity configurations.^38,39^ Also, the local crystal field influences both the energy levels and the electric dipole moments with components of distinct parities.^40^ While first-principles calculations are challenging for a real sample, in the following sections we develop two alternative, self-referenced, experimental strategies for obtaining the parameter *C* based on the thermal population distribution of the TC levels.

To develop the self-referenced procedures, we first need to assume that the particles are in direct thermal contact with a heating/cooling element, which heat transfer rate *Q̇* can be controlled. The rate *Q̇* can also be represented by an external parameter *P*, for instance, as the position of the control knob for the electrical power dissipated in a resistive heater or in a Peltier module, provided that the relation between *P* and *Q̇* is linear. The heat source will produce temperature gradients across the composite system (particles + surroundings), depending on the thermal resistances of the materials. The exact temperature at the location of the particles is not necessarily known beforehand, but for suitable temperature ranges it will be proportional to the rate *Q̇* (see Section S1 of the Supporting Information (SI) material for a detailed analysis). In other words, the temperature of the particle, *T*, varies linearly with the heat transfer rate *Q̇*, consequently also with the control parameter *P*, as

2 $$T=aP+T_{0}$$

where *a* indicates the effectiveness of the control knob in tuning the temperature, being determined by the thermal resistance and boundary conditions of the system. *T*_0_ corresponds to the equilibrium temperature of the thermometer when the control system is off. When the system’s effective thermal resistances change with temperature, a relation as eq 2 should also be verified around a smaller temperature range of interest. We remark that our methodological proposal depends only on the existence of a linear relation between *T* and *P* for a given region used for the self-referenced calibrations, but not on the specific values of the constants *a* and *T*_0_.

In this work, the heating device is a closed-cycle cryostat that allows tuning the electrical power applied to its internal resistive elements. It acts to increase the temperature inside the cryocooler chamber above the liquid helium boiling point (4.2 K). Since in our case we can directly tune the electrical power delivered by the resistive elements, we can make *P* ∝ *Q̇*. Section S2 of the SI shows the measured linearity between *P* and the cryostat internal temperature, measured with an independent thermometer (see the details in the Materials and Methods section). Given the validity of eq 2, we discuss in what follows that *C* can be obtained by studying the relation between *R*_Stark_ and *P* at sufficiently high temperatures and also by exploiting the inflection point in the optimal sensitivity region.

#### Self-Referenced Calibration Using the High Temperature Limit

For thermal energies much higher than the energy difference between the TC sublevels (*k*_B_ *T* ≫ Δ*E*), the occupation probability asymptotically becomes 50% for both, which can be used to estimate *C*. In this case, exp­(−Δ*E*/*k*_B_ *T*) ≈ 1, so *R*_Stark_ ≈ *C* = *g*_2_ *A*_20_/*g*_1_ *A*_10_.

If *C* or Δ*E* do not change significantly at high temperatures, it is possible to experimentally extrapolate the asymptotic limit for *R*_Stark_(*P*) from measurements at sufficiently large temperatures, therefore extracting *C*. This is achieved by measuring a set of spectra for various control parameters *P*, obtaining a *R*_Stark_(*P*) curve. Although *R*_Stark_(*P*) has a complicated dependence with *P*, a better experimental relation would be obtained by taking the product *P* ln *R*_Stark_ = *P* ln *C* – Δ*E*/*k*_B_ × *P*/(*aP* + *T*_0_). While this expression seems too complex, a plot of *P* ln *R*_Stark_ *vs P* is such that the slope at a given point can be written as

3 $$\frac{d\left(P\cdot \ln R_{\mathrm{Stark}}\right)}{dP}=\ln C-\frac{\Delta E}{k_{B}T}\frac{T_{0}}{T}$$

That is, the asymptotic slope of *P* · ln *R*_Stark_ in the high-temperature limit can be used to extract ln *C*. Notice that the curve can be represented directly using the experimental data and we have not referred to the sample’s temperature in any step, neither is necessary to determine *a* or *T*_0_. Thus, even though the thermal resistances of the system may change with *T*, our proposal remains valid to extract *C* as long as *C* itself does not change significantly with temperature. The linearized parameters *a* and *T*_0_ will change with the calibration mean set point temperature, but they do not influence significantly the estimate of *C* around sufficiently large temperatures *T*. Since the temperature-dependent term in eq 3 scales with *T* ^–2^, the linear slope can be attained for relatively low temperatures provided that Δ*E*/*k*_B_ ≪ *T* and *T*_0_ ≪ *T*. Also notice that the *T* ^–2^ dependence suggests that eq 3 converges faster to ln *C* instead of using ln *R*_Stark_ only.

Performing the measurements at high temperature ranges reduces inaccuracy, but clearly the thermal stability of the host matrix limits the maximum temperature it supports without changing its properties (including possible phase transitions at high temperatures). Therefore, the energy difference between the TC Stark sublevels should be low enough to allow reaching the “high” temperature limit without significant changes in the host matrix. Naturally, this limits the range of optimal thermal response,^37^ making this self-referenced calibration procedure very efficient to thermometers at cryogenic temperature ranges. From the practical point-of-view, at higher temperatures blackbody radiation might intrude and may need to be properly subtracted. Stark lines will spectrally broaden and might not be as easily separable. Intruding bands can also be thermally activated and thus disrupt the *R*_Stark_ measurement.^31^

To demonstrate this strategy, we placed the Y_2_O_3_: (1.5%) Yb^3+^/(0.5%) Er^3+^ nanoparticles inside a heating/cooling device (cryostat). The sample was excited with laser illumination at 980 nm, and the luminescence emission in the NIR, around 1550 nm, was collected (see Materials and Methods section). This luminescence band corresponds to transitions between the Stark sublevels of the ^4^I_13/2_ manifold of the Er^3+^ ions to the Stark sublevels of the ground-state manifold (^4^I_15/2_).^16^ In particular, we used the luminescence emission of the two lowest Stark sublevels of ^4^I_13/2_, labeled as Y_1_ and Y_2_.^41^ These sublevels are separated by only 33.08 ± 0.05 cm^–1^ at 20 K (see Section S3 of the SI), thus making the optimum temperature measurement window^37^ of 14–24 K. The simplified energy-level structure of the ^4^I_13/2_ manifold is shown in Figure 1a. The resulting observed luminescence spectra after laser excitation at 980 nm are shown in Figure 1b for heat transfer rates from 0 up to 1.563 W (the maximum the device can provide). This power range corresponds to internal temperature variations between 4.4 and 350.0 K.

**Figure 1:**
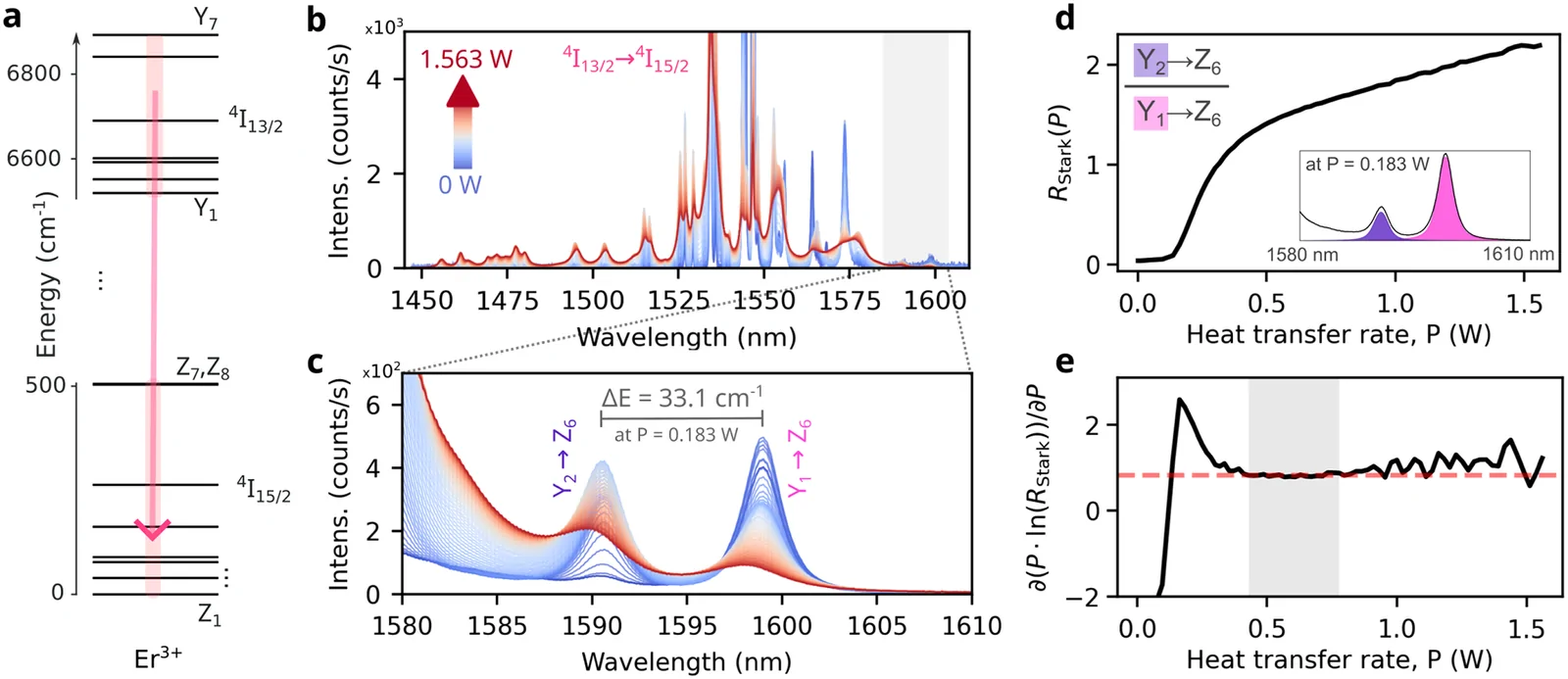
**a**: Simplified energy-level diagram of Er^3+^ ions in Y_2_O_3_ matrix. The arrow and shaded regions represent spontaneous radiative decay from all Stark sublevels of ^4^I_13/2_ to those of ^4^I_15/2_. **b**: Resulting luminescence spectra of the transitions as a function of the heat transfer rate, *P. Y*-axis was limited to improve reading. **c**: *P* sweep of the two particular Stark–Stark lines (Y_1_ → Z_6_ and Y_2_ → Z_6_) chosen as thermometric probes. The energy centers of the lines were measured at an arbitrary *P* point according to the methods described in Section S3. **d**: Measured *R*_Stark_ as a function of *P*, for the full working range of the cooling/heating device (cryostat). Inset shows a spectrum at an arbitrary *P* point with the Voigt fittings of the Stark lines used to calculate *R*_Stark_. **e**: Derivative of the experimental curve *P* · ln *R*_Stark_, demonstrating linearity in the limit of high temperatures. The shaded region shows the limits used to calculate the average of the points (red dashed line).

We selected the Stark pair Y_1_ → Z_6_ and Y_2_ → Z_6_, located between 1585 and 1605 nm, because they are well separated from neighboring peaks (Figure 1c). This spectral isolation enables unambiguous transition assignment and reliable Voigt-profile fitting (see Section S3 of the SI for details on the deconvolution procedure). Additionally, this wavelength range lies within the telecommunication L band, and also in the NIR III optical window for biological tissues, which can be important to a variety of applications.

In Figure 1d we show the *R*_Stark_(*P*) curve for the full working range allowed by the heating system. Based on eq 3, we can determine ln *C* by the slope of the *P* · ln *R*_Stark_ *vs P* curve in the asymptotic limit of *P* → ∞. Figure 1e depicts the first derivative of *P* · ln *R*_Stark_ with respect to *P*, showing that for *P* → ∞, the curve resembles a straight line. To extract ln *C*, we used an average of 22 data points of Figure 1e between *P* = 0.43 W and *P* = 0.78 W (shaded region) where *R*_Stark_ presents a reasonable signal-to-noise ratio. We therefore obtain *C* = 2.3 ± 0.1. For higher *P* values, the overall intensity of the luminescence lines decreases while their width increases, making spectral separation more difficult thus increasing uncertainty. We notice that for *P* > 0.78 W, the curve derivative slightly increases, most probably due to the low quality of the spectral separation. The uncertainty was calculated based on conventional error propagation from the fitting (see Section S5 of the SI). This value is in agreement with that determined by temperature-dependent verification (Figure S4b).

By measuring *R*_Stark_ and Δ*E* for a given spectrum, and considering *C* = 2.3 ± 0.1, one has all necessary parameters to obtain *T* from eq 1. In Figure 2a we show the measured thermodynamic temperatures and the nominal ones, determined with an independent probe (the cryostat internal thermometer) for validation. The right-hand axis shows the difference between the temperature measured using the method just described and the cryostat reference. This represents the accuracy of our thermometer, since the cryostat manufacturer guarantees an accuracy of ±10 mK around 30 K.^42^ Our optical thermometer shows a maximum accuracy of ±0.5 K for temperatures within its optimum window. In Figure 2b we show the relative sensitivity *S*_*r*_(*T*) = *R* ^–1^ *∂R*/*∂T* and the thermal uncertainty σ_*T*_(*T*) (resolution), calculated based on uncertainty propagation (see Section S5 of the SI). *S*_*r*_(*T*) and σ_*T*_(*T*) are adequate parameters to compare different types of thermometers. Within the optimum window, the thermal resolution is ±0.5 K and the relative sensitivity ranges between 25% K^–1^ and 10% K^–1^, typical values for temperatures in cryogenic ranges. For higher temperatures, σ_*T*_ increases since it scales with $S_{r}^{-1}$ . For lower temperatures, σ_*T*_ also increases because the signal-to-noise ratios of the luminescence signals decrease.

**Figure 2:**
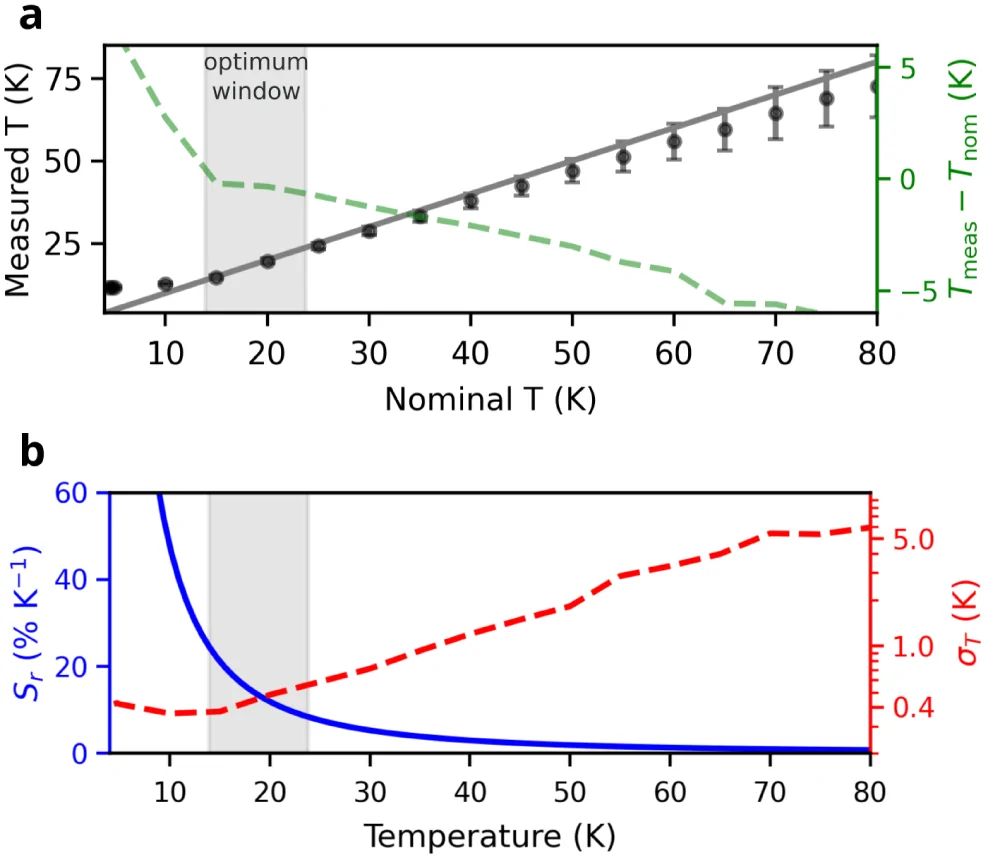
**a**: Comparison between temperature measurements of the proposed optical thermometer (measured T) and the internal cryostat high-resolution reference (nominal T). The solid straight line represents the function *y* = *x*. The right-hand *y*-axis shows the difference between these two quantities. **b**: Characterization of the optical nanothermometer showing the relative sensitivity (*S_r_*) and thermal uncertainty (σ*_T_*).

#### Self-Referenced Calibration Using the LIR Curve Inflection Point

The function *R*_Stark_(*T*) [eq 1] exhibits an inflection point at *T*_infl_ = Δ*E*/2*k*_B_, corresponding to the temperature where the absolute sensitivity *S*_*a*_ = *∂R*_Stark_/*∂T* reaches its maximum. It is the upper bound of the optimum temperature measurement window $\left[\Delta E/\left(2+\sqrt{2}\right)k_{B},\Delta E/2k_{B}\right]$ .^37^

Similarly, the function *R*_Stark_(*P*) will also exhibit an inflection point at *P*_infl_ = (*T*_infl_ – *T*_0_)/*a*. This occurs because *∂* ^2^ *R*/*∂P* ^2^ = *a* ^2^ · *∂* ^2^ *R*/*∂T* ^2^. By acquiring a set of spectra for a range of *P* values around *P*_infl_, one can locate *R*_Stark_(*P*_infl_). At this point, *T* = *T*_infl_, allowing the inversion of eq 1 to determine *C* as *C* = *R*_Stark_(*P*_infl_) · e^2^. This approach requires that the linear *T*(*P*) relation remains valid within a suitable range around *P*_infl_. Notably, knowledge of *a* and *T*_0_ is unnecessary, as the calibration parameter *C* depends solely on identifying *R*_Stark_(*P*_infl_).

This identification can be done, for instance, by second-order differentiation of the experimental data with regard to *P*. A common technique is Savitzky–Golay filtering,^43^ which fits local subsets of data points with low-order polynomials using linear least-squares and applies the result via convolution. The individual polynomials are then differentiated individually. Increasing the number of points improves smoothing but introduces bias, potentially shifting the apparent inflection point away from *P*_infl_.^44^

Furthermore, since the FWHM of the *S*_*a*_ curve scales linearly with Δ*E*, the uncertainty in obtaining its maximum also increases proportionally. Therefore, the accuracy of thermometers operating optimally in higher temperatures (high Δ*E*) will be lower.

We experimentally demonstrate the feasibility of finding the inflection point using the Savitzky-Golay differentiation for two independent pairs of TC Stark sublevels: (i) E_1_ and E_2_ from the ^4^S_3/2_ manifold, resulting in emission lines in the green spectral range,^17^ and (ii) Y_1_ and Y_2_ from the ^4^I_13/2_ manifold resulting in emission lines in the NIR spectral range.^16^ The luminescence lines from E_1_ and E_2_ sublevels to the ground state are shown in Figure 3, while those from Y_1_ and Y_2_ were already presented in Figure 1b. Section S4 of the SI shows the temperature-dependence characterization for the green spectral band. In particular, the energy difference between E_1_ and E_2_ is $\Delta E_{E_{1}E_{2}}=86.6\pm 0.2{\;\mathrm{cm}}^{-1}$ at 50 K, thus making the optimum temperature measurement window 36–62 K.

**Figure 3:**
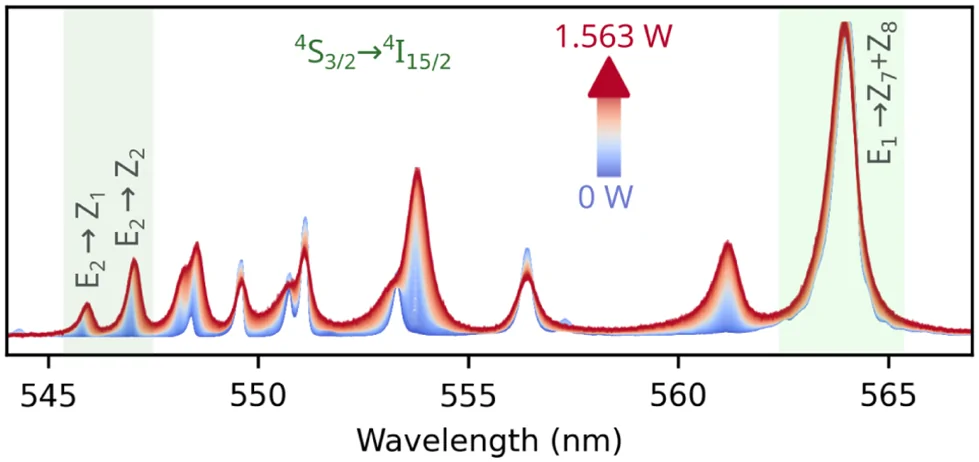
Sweep of the resulting luminescence spectra of the transition ^4^S_3/2_ → ^4^I_15/2_ as a function of the heat transfer rate, *P*. Counts are normalized to the maximum peak at 564 nm for visualization. The count rate of the maximum peak is 80 counts/s at room temperature. The shaded spectral areas highlight the lines used for absolute temperature measurements.

Figure 4a shows *R*_Stark_(*P*) for E_1_ and E_2_ around *P*_infl_, while Figure 4b presents its first and second derivatives. The derivatives of the experimental curve were computed using a second-order Savitzky-Golay filter with 40 points per subset. A total of 100 spectra has been acquired around *P*_infl_. The curve exhibits an inflection point at *P*_infl_ = 0.309 ± 0.001 W. We then measure *R*_Stark_(*P*_infl_) = 0.035 ± 0.002 to obtain *C* = 0.261 ± 0.015, in agreement with the value determined independently in Figure S7b (See Section S4 of the SI). By measuring *R*_Stark_ and Δ*E* for a given spectrum, and considering *C* = 0.261 ± 0.015, one has all necessary parameters to obtain *T* from eq 1. Figure 4c shows the measured temperatures, plotted against the nominal temperatures. Excellent agreement is observed within the optimum range, with temperature accuracy better than 0.5 K. In Figure 4d we show the relative sensitivity and the temperature uncertainties calculated based on error propagation (Section S5 of the SI). The minimum uncertainty is 0.7 K, while relative sensitivities as high as 9.0% K^–1^ have been obtained in the optimum window.

**Figure 4:**
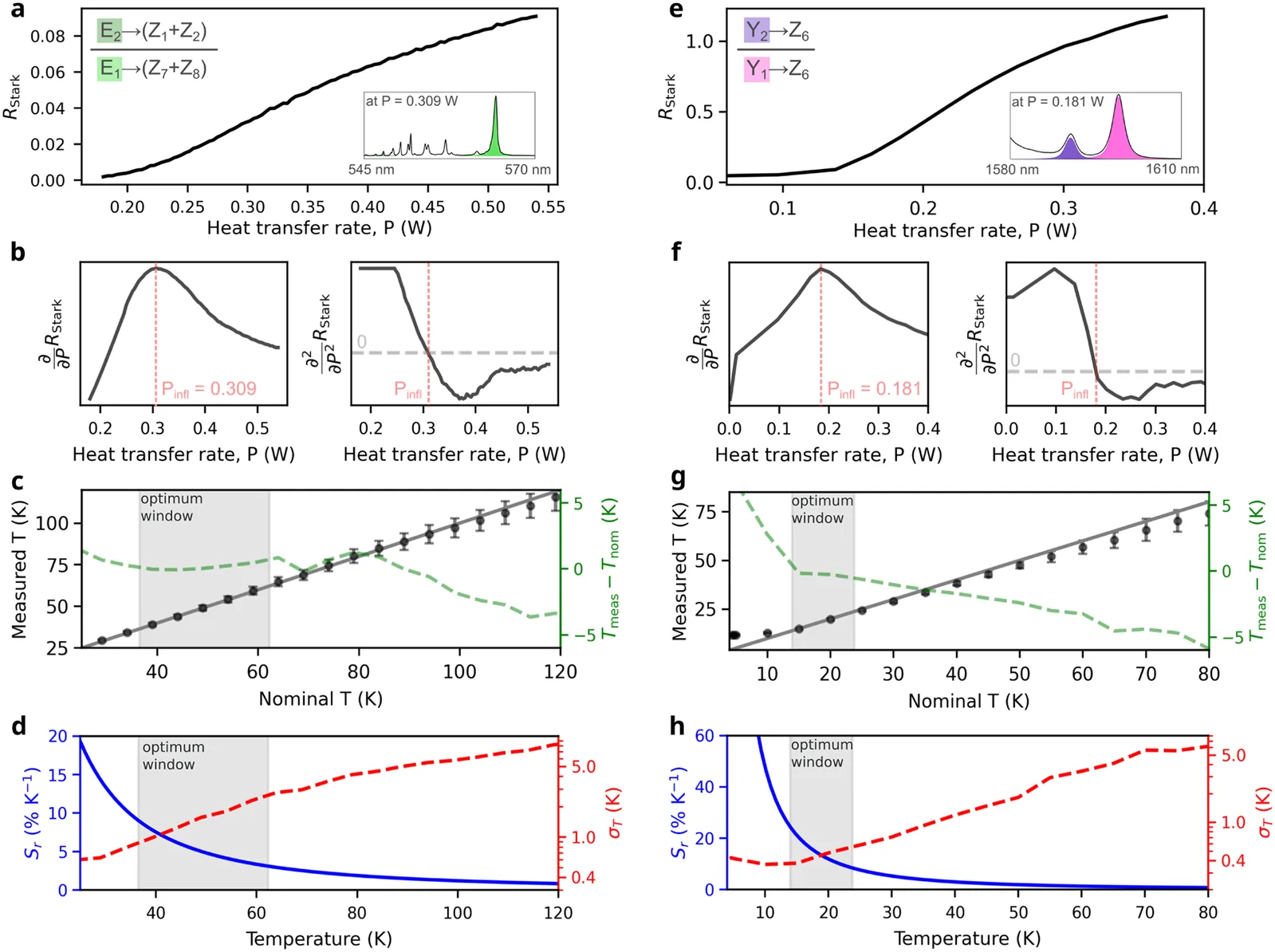
Self-referencing via the inflection point method. Left column by using E_1_ and E_2_ Stark sublevels of ^4^S_3/2_ and right column by using Y_1_ and Y_2_ Stark sublevels of ^4^I_13/2_. **a** and **e**: *R*_Stark_ curves as a function of the external control parameter. Insets show the spectra at *P*_infl_ with the Voigt fittings of the Stark lines used. **b** and **f**: first and second derivatives of the respective experimental curves obtained with a Savitzky-Golay algorithm. The horizontal dashed lines on the second derivative plots mark where it is zero. **c** and **g**: Comparison between temperature measurements through the luminescence spectra (measured T) with the internal cryostat references (nominal T). The solid straight lines represent the function *y* = *x*. The right-hand *y*-axes show the difference between these two quantities. **d** and **h**: Characterization of the optical nanothermometers showing the relative sensitivities and thermal uncertainties.

Figures 4e–h present analogous results for *R*_Stark_(*P*) obtained from the Y_1_ and Y_2_ Stark sublevels of the ^4^I_13/2_ manifold. We obtained 17 spectra around *P*_infl_. The second-order Savitzky-Golay filter uses 6 points per subset. In this case, we obtain *P*_infl_ = 0.181 ± 0.001 W, and *C* = 2.27 ± 0.06, in agreement with that obtained with the previous method of calibration (*C* = 2.3 ± 0.1), and also in agreement with that determined through temperature-dependent verification in Figure S4b. Similarly, we observe accuracies better than 0.5 K in the optimum window and a minimum uncertainty of 0.4 K. The maximum relative sensitivity in the optimum window is 25% K^–1^.

## Discussion

We demonstrate robust absolute primary optical nanothermometric measurements using individual Stark sublevels in a rare-earth-doped solid. We developed two independent self-referenced calibration procedures that do not rely on external thermometric probes for the thermodynamic temperature determination: 1) exploiting the high-temperature limit of the *P* · ln *R*_Stark_(*P*) curve, enabling absolute thermometry optimally in the cryogenic regime; and 2) identifying the inflection point of the *R*_Stark_(*P*) curve, which occurs at *T*_infl_ = Δ*E*/2*k*_B_. To validate both approaches, we used Yb^3+^,Er^3+^-codoped yttrium oxide (Y_2_O_3_) nanoparticles, which exhibit well-resolved Stark spectral lines. The precise design of the host matrix is essential to ensure a sufficiently large Stark splitting and narrow spectral line widths. In particular, the choice of yttria (Y_2_O_3_) as host matrix allows to observe Stark–Stark transitions with line widths of 0.8 nm, enabling individual lines to be clearly spectrally resolved, identified and integrated.^17,45^

Two independent Stark sublevel pairs were investigated: E_1_/E_2_ from the ^4^S_3/2_ manifold and Y_1_/Y_2_ from the ^4^I_13/2_ manifold. The former pair produces green luminescence and enables absolute thermometry with an accuracy of 0.5 K within the optimal range of 36–62 K, whereas the latter emits in the NIR and achieves 0.5 K accuracy in the 14–24 K range. Despite the fact that green light is easier to detect, near-infrared light is more adequate to the study of biological samples. In any case, other Stark sublevel pairs can be selected provided that their spectral lines are spectrally resolvable, and that the thermalization dynamics follows Boltzmann statistics. This allows operation over different temperature ranges, including possibly near room temperature.

A central assumption of this work is that eq 1 remains valid over a suitable temperature window. This translates into conditions such as 1) Δ*E* and *C* are temperature-independent; 2) no additional photophysical processes effectively compete with electron–phonon thermalization; and 3) the excitation power is low enough to avoid non-negligible self-heating. Regarding condition 1): The radiative decay rates of Ln^3+^ systems are known to exhibit slight temperature dependence, but in most cases this variation is negligible,^37,46^ allowing *C* to be approximated as temperature-invariant. Likewise, matrix conformational changes with temperature can modify the Stark splitting structure, thus altering Δ*E*. In the SI material, we presented Δ*E* vs *T* measurements showing variations on the order of 3 × 10^–3^ cm^–1^/K; as a result, Δ*E* can also be considered as temperature-invariant depending on the targeted accuracy of the absolute thermometer. Concerning conditions 2) and 3): Competing photophysical processes such as cross-relaxation or excited-state absorption would modify the LIR *vs T* curve, deviating it from the simple exponential dependence in eq 1.^36^ Similarly, self-heating – more pronounced at lower temperatures for a fixed laser power – would also distort *R*_Stark_ from eq 1. To avoid these artifacts, we carefully controlled the synthesis route, doping concentration and size of the nanoparticles during chemical assembly,^17^ as well as the excitation power densities during experiments. The controlled temperature-dependent measurements of *R*_Stark_, presented in Sections S3 and S4 of the SI, ensure that *R*_Stark_ follows a plain Boltzmann distribution over a wide temperature range, with the correct *C* and Δ*E* parameters.

Single-particle measurements have been applied for relative primary nanothermometry,^23,31,45^ but it has been shown that different particles can have slightly different calibration parameters, even stemming from the same batch.^23,27,28^ Therefore, using the methods developed in this work for single nanoparticles would allow, for instance, determining the local thermodynamic temperature in the nano- or microscale without disturbing its behavior with an external probe, specially in environments with multiple heat sources or sinks, as, for instance in *in vivo* biological samples.^47^ For example, a contactless absolute nanothermometer can be used to eliminate several artifacts, possibly helping to address current discrepancies in single cell thermometry.^48–50^ Furthermore, because the thermalization of rare-earth ions with their local environment occurs at the single-ion level, single-ion thermometry could provide a route toward absolute temperature measurements using quantum emitters.^6^ Precision can be further enhanced when single-qubit thermometers exploit quantum coherence and entanglement.^51^ Such capabilities open new opportunities for quantum thermometry and quantum thermodynamics investigations, for example in probing heat transport and dissipation in nanoscale subkelvin electronic circuits.^52,53^

It is worth mentioning that the approach developed in this work is not suitable if one uses the full manifold-to-manifold intensity band to calculate the LIR (*R*_manifold_) because the energy difference governing Boltzmann equation [eq 1] should be treated as an effective value, which depends not only on the energy-level structure of the Ln^3+^ ion, but also on all radiative decay rates of the lines comprising the luminescence band.^36^ This effective energy difference is different from the barycenter energy difference,^17^ meaning that it is not possible to extract it solely from the luminescence spectrum without a previous temperature calibration.

## Materials and Methods

### Experimental Details

We employed a powder sample of Y_2_O_3_: Yb^3+^ (1.5 mol %)/Er^3+^ (0.5 mol %) nanoparticles with an average diameter of 80 nm. Sample’s characterization details as morphology and X-ray diffractogram can be found in ref 17.

The dry powder was pressed into a copper sample holder and mounted inside a closed-cycle cryostat (Cryostation s50, Montana Instruments). An internal temperature sensor (Cernox CX-1070, Lake Shore Cryotronics) serves as a reference for comparison. The manufacturer calibration curve displays a typical accuracy of ±6 mK at 10 K and ±48 mK at 300 K.^42^ By tuning the heat transfer rate (input power), the internal cryostat temperature can be changed from 4.4 to 350.0 K, according to the internal probe.

The sample was excited by a femtosecond laser source emitting at 980 nm with 80 MHz repetition rate and 7 nm of spectral pulse width. The light excitation irradiance was 2.0 kW/cm^2^. The Yb^3+^ ions work as sensitizers and the observed luminescence spectra stem from electronic transitions of the Er^3+^ ions.^23^ We performed measurements for both green and NIR spectral regions. The green luminescence spectrum originates from an upconversion process via a well-known Yb^3+^ → Er^3+^ two-photon energy transfer mechanism,^23^ whereas the NIR emissions arises primarily from nonradiative relaxations from ^4^I_11/2_ to ^4^I_13/2_ after a single-photon energy transfer process.^16^ Emission was collected in reflection geometry using a lens with 35 mm focal length. The resulting emitted light was directed to a spectrometer with a high-resolution grating (1800 grooves/mm) and detected by a cooled CCD camera. Integration time for the green and NIR spectrum was 60 and 20 s, respectively. Further experimental details, including a diagram of the experimental setup, can be found in a previous work from our group, ref 17.

## Supplementary Material



## Data Availability

The data that support the findings of this study are available from the corresponding author upon reasonable request.

## Notes

The temperature dependence of the material’s luminescence spectra in the green and NIR spectral ranges is presented in the Supporting Information material. A detailed analysis of the linearity between temperature and the heating device’s power input is also provided, together with a discussion of accuracy versus precision in the context of the methods employed in this work (PDF)
