# Supplementary material for: Absolute Primary Nanothermometry Using Individual Stark Sublevels of Rare-Earth-Doped Crystals
Source: J Am Chem Soc. 2026 Sep 27;148(39):42847–55. doi: 10.1021/jacs.6c17449 (PMC13651606; doi:10.1021/jacs.6c17449)
Supplement: Supplementary file 1 [file ja6c17449_si_001.pdf]

Supporting Information: Absolute Primary Nanothermometry Using Individual Stark Sublevels of Rare-Earth-doped Crystals

Allison R. Pessoa,*,†,‡ Thomas Possmayer,¶ Jefferson A. O. Galindo,† Luiz F. dos Santos,§ Rogéria R. Gonçalves,§ Leonardo de S. Menezes,¶,† and Anderson M. Amaral†

†*Department of Physics, Universidade Federal de Pernambuco, 50740-540 Recife-PE, Brazil*

‡*Federal Institute of Education, Science and Technology of Pernambuco, 50740-545 Recife-PE, Brazil*

¶*Chair in Hybrid Nanosystems, Faculty of Physics, Ludwig-Maximilians-Universität München, 80539 München, Germany*

§*Department of Chemistry, Center of Nanotechnology and Tissue Engineering- Mater Lumen Laboratory, Faculty of Philosophy, Science and Letters of Ribeirão Preto, University of São Paulo, 14040-901 Ribeirão Preto-SP, Brazil.*

E-mail: allison.pessoa@ufpe.br

Contents

S1 Relation between the temperature of the particle and the heat transfer rate **S2**

S2 Linear relation between P and the cryostat internal temperature **S4**

S3 Characterization of the Stark lines $\mathbf{Y}_1 \rightarrow \mathbf{Z}_6$ and $\mathbf{Y}_2 \rightarrow \mathbf{Z}_6$ **S4**

S4 Characterization of the Stark lines $\mathbf{E}_1 \rightarrow (\mathbf{Z}_7 + \mathbf{Z}_8)$ and $\mathbf{E}_2 \rightarrow (\mathbf{Z}_1 + \mathbf{Z}_2)$ **S6**

S5 Accuracy and precision **S9**

References **S10**

S1 Relation between the temperature of the particle and the heat transfer rate

The thermal interface between a heating/cooling element and a nanoparticle thermometer, as depicted in Fig. S1**a**, can be modeled by a thermal circuit shown in Fig. S1**b**. The heat source delivers heat at the rate $\dot{Q}$. Fourier's law of heat conduction leads to an analogy to Ohm's law for electrical circuits:[S1] $\Delta T = R \cdot \dot{Q}$, where ΔT is the temperature difference between two points in space. ΔT is the analogous of voltage while $\dot{Q}$ is the analogous of electrical current. R is the thermal resistance of the material, or at interfaces.

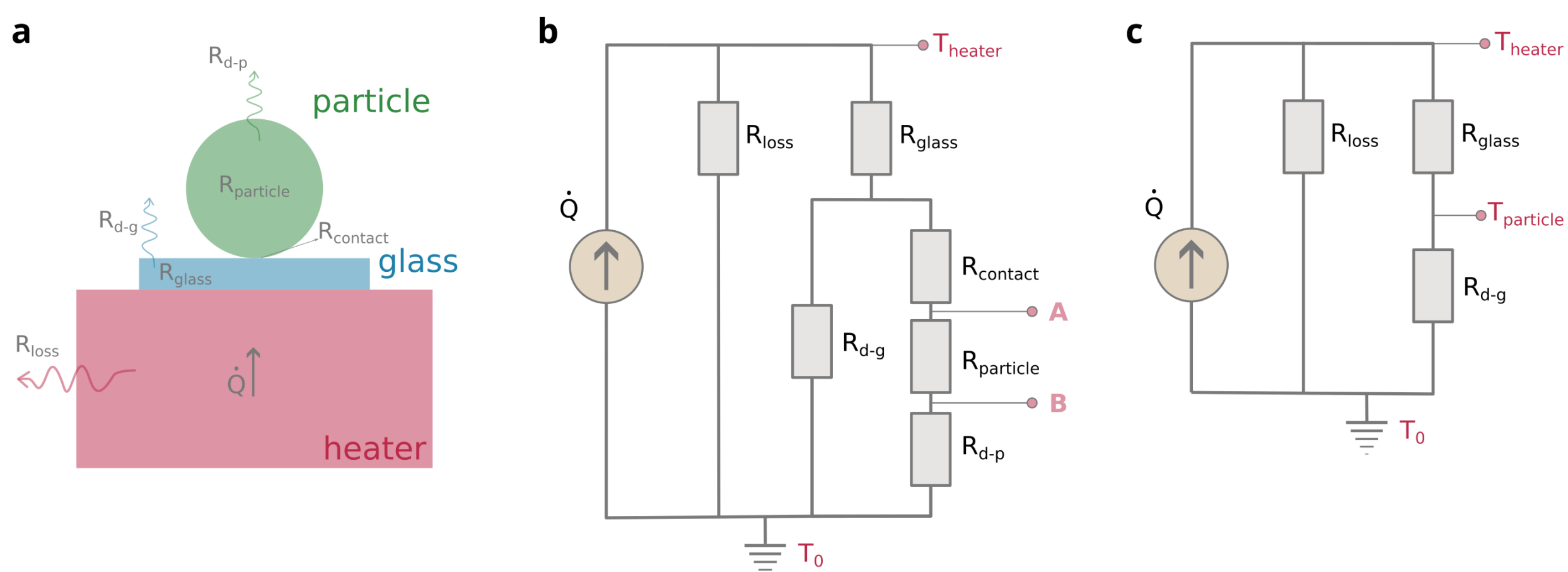

Figure S1: **a**: Schematics of the interface between a heater element and a nanoparticle thermometer. **b**: Thermal circuit modeling the situation presented in **a**. **c**: Simplification of the thermal circuit presented in **b** to allow easier physical interpretation.

The heater dissipates power to its surroundings with resistance R_{loss} and to the glass + particle system with equivalent resistance $R_{\text{glass}} + R_{\text{d-g}}(R_{\text{contact}} + R_{\text{particle}} + R_{\text{d-p}})/(R_{\text{d-g}} + R_{\text{contact}} + R_{\text{particle}} + R_{\text{d-p}})$. Here, R_{glass} is the thermal resistance of the glass, $R_{\text{d-g}}$ is the thermal resistance representing the heat dissipation by the glass through its surface, R_{contact} is the thermal contact resistance between the particle and the glass, R_{particle} is the thermal resistance of the nanoparticle, and $R_{\text{d-p}}$ is the thermal resistance representing the heat dissipation by the particle.

Due to the nanoscale size of the particle, $R_{\text{particle}} \ll R_{\text{d-p}}$ (small Biot numbers[S1]), meaning that the temperature inside the particle can be considered uniform. So, either point A or B in Fig. S1**b** can represent the temperature of the particle ($R_{\text{particle}} \to 0$). Furthermore, the heat dissipated through the glass is much greater than the heat dissipated through the particle, meaning

that $R_{\text{d-g}} \ll R_{\text{d-p}}$ and $R_{\text{d-g}} \ll R_{\text{contact}}$. As a result, we can simplify the thermal circuit to that presented in Fig. S1**c**, only as means for obtaining easier physical understanding. In this case, T_{ptc} is written as

$$T_{\text{ptc}} = T_0 + \frac{R_{\text{d-g}} \cdot R_{\text{loss}}}{R_{\text{glass}} + R_{\text{d-g}} + R_{\text{loss}}} \dot{Q} \,, \tag{S1}$$

thus showing that $T = aP + T_0$, with $P \propto \dot{Q}$ should be a valid prescription to describe our experimental system. T_0 can be the room temperature, for instance, or the helium boiling point temperature if the system is placed inside a cryostat, as it is the case of the present study. It is worth noticing that any configuration of heat or temperature sources with linear thermal resistors would lead to the same linear result, $T = aP + T_0$, since it is a fundamental consequence of the the heat equation $\nabla^2 T = 0$ in steady state and homogeneous materials, provided that, for suitable temperature ranges, we consider that the thermal resistances are temperature-independent.

Even if the thermal resistances change with the temperature, the linear relation should still be verified for a suitable range around a given temperature point T_c. Using the Norton equivalent circuit for Fig. S1**c**, we can write $T_{\text{particle}} = T_0 + R_{eq}(T_{\text{particle}})\dot{Q}$. For small temperature variations around T_c, $T_{\text{particle}} = T_c + \delta T$, $\dot{Q} = \dot{Q}_c + \delta\dot{Q}$ and $R_{eq} = R_c + R'\delta T$, such that

$$\delta T = \frac{R_c \delta \dot{Q}}{1 - R'\dot{Q}} \quad . \tag{S2}$$

For $\dot{Q}_c \gg \delta\dot{Q}$,

$$\delta T = \frac{R_c \delta \dot{Q}}{1 - R'\dot{Q}_c} \quad . \tag{S3}$$

Thus, the overall relation $T = aP + T_0'$ should be valid in a small neighborhood close to the calibration temperature T_c as long as there are no abrupt changes in the thermodynamics properties of the thermal circuit elements (such as a phase transition). Moreover, notice that the offset temperature $T_0' \neq T_0$ in general due to the temperature dependence of the thermal resistance, R'.

S2 Linear relation between P and the cryostat internal temperature

The cryostat allows controlling the input power of its internal heaters to increase the temperature above the helium boiling point. In the main text, we assumed that the relation between the input power and the internal temperature is linear for a suitable temperature range. Figure S2 shows this relation measured for the complete system's working range.

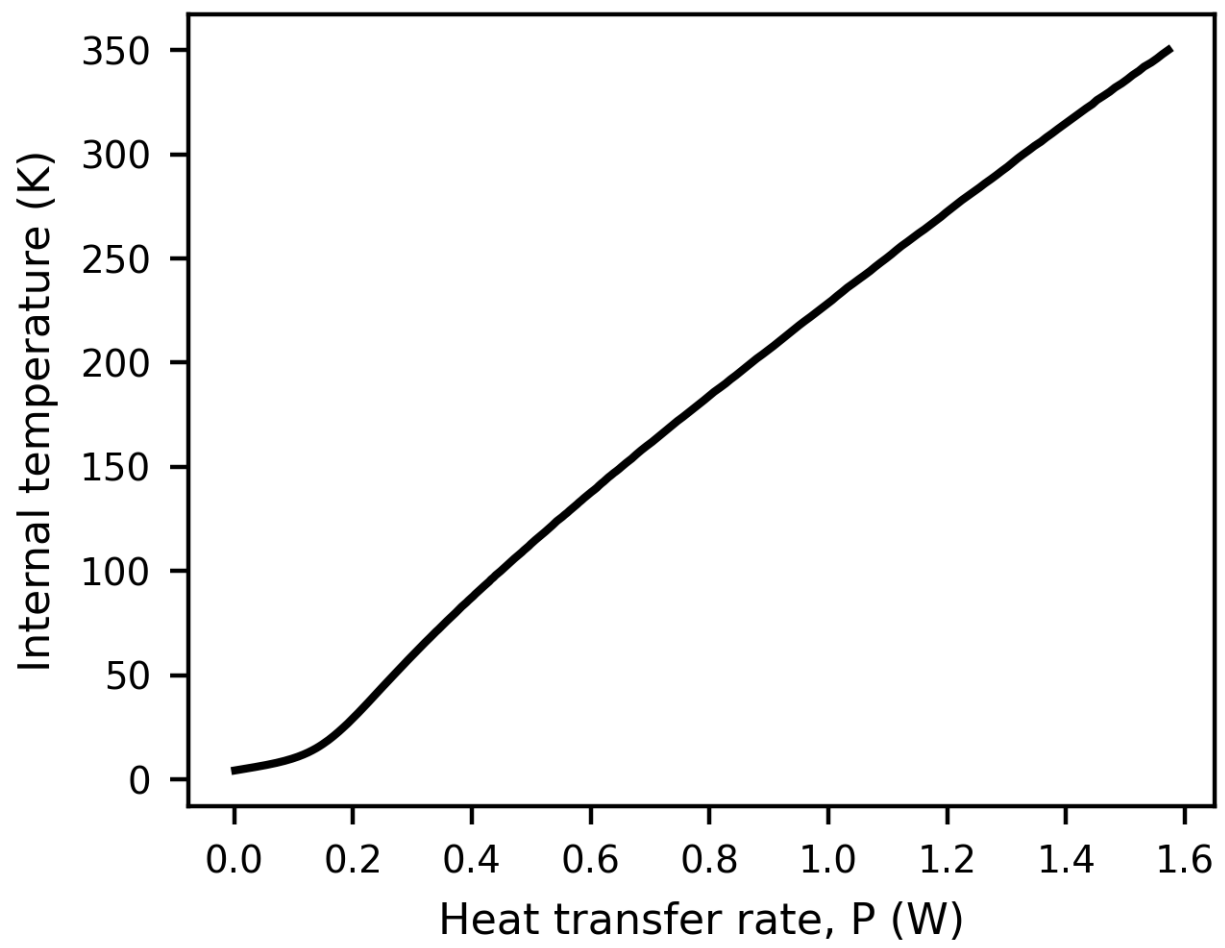

Figure S2: Measured relation between cryocooler input power (energy transfer rate) and its internal temperature.

S3 Characterization of the Stark lines $\mathbf{Y}_1 \rightarrow \mathbf{Z}_6$ and $\mathbf{Y}_2 \rightarrow \mathbf{Z}_6$

The Stark sublevels Y_1 and Y_2 belong to the $^4I_{13/2}$ manifold of the Er^{3+} ions, as depicted in Fig. 1**a** of the main manuscript. They are used as pair of thermally coupled sublevels for absolute primary thermometry measurements by monitoring the lines $Y_1 \rightarrow Z_6$ and $Y_2 \rightarrow Z_6$, where Z_6 is a given Stark sublevel of the ground state manifold ($^4I_{15/2}$).

The selected Stark lines were deconvolved from the luminescence spectrum using a Voigt profile fitting, according to the procedures detailed in ref. [S2]. The partially superposed emission lines were fitted with the function

$$V(x; V_0, x_0, \sigma, \gamma) = V_0 \int_{-\infty}^{\infty} G\left(x'; \sigma\right) L\left(x - x'; \gamma\right) \mathrm{d}x' \,, \tag{S4}$$

where $G(x; x_0, \sigma) = \frac{1}{\sigma\sqrt{2\pi}} \exp\left(-\frac{(x-x_0)^2}{2\sigma^2}\right)$ is the Gaussian component and $L(x; x_0, \gamma) = \frac{\gamma/\pi}{(x-x_0)^2+\gamma^2}$

is the Lorentzian component. V_0, x_0, σ and γ are fitting parameters.

Fig. S3**a** shows a luminescence spectrum in the wavelength range of interest along with the individual contributing Voigt profiles. The sum of all Voigt peaks composes the fitted spectrum. The residue of the fitted spectrum is shown in Fig. S3**b** and they are smaller than 4 % in the whole spectral range considered. Since the transitions $Y_1 \rightarrow Z_6$ and $Y_2 \rightarrow Z_6$ share the same final state (Z_6), the difference between their energy barycenters is equal to the 'real' energy difference between Y_1 and Y_2 states ($\Delta E_{\text{bary}} = \Delta E_{Y_1 Y_2}$). To calculate ΔE_{bary} one needs to transform the spectral lines from the wavelength representation $I(\lambda)$ to the energy representation $\tilde{I}(E)$, then obtain the individual barycenters by $E_{\text{bary}} = \left(\int_{-\infty}^{\infty} E \cdot \tilde{I}(E) \mathrm{d}E\right) / \left(\int_{-\infty}^{\infty} \tilde{I}(E) \mathrm{d}E\right)$, as described in ref. [S3]. Fig. S3**c** shows the measured $\Delta E_{Y_1 Y_2}$ according to this procedure as a function of the internal cryostat temperature, for characterization. As the temperature increases, the host lattice expands changing the effective crystal field on the Er^{3+} ions, hence shifting the energy of the Stark sublevels.

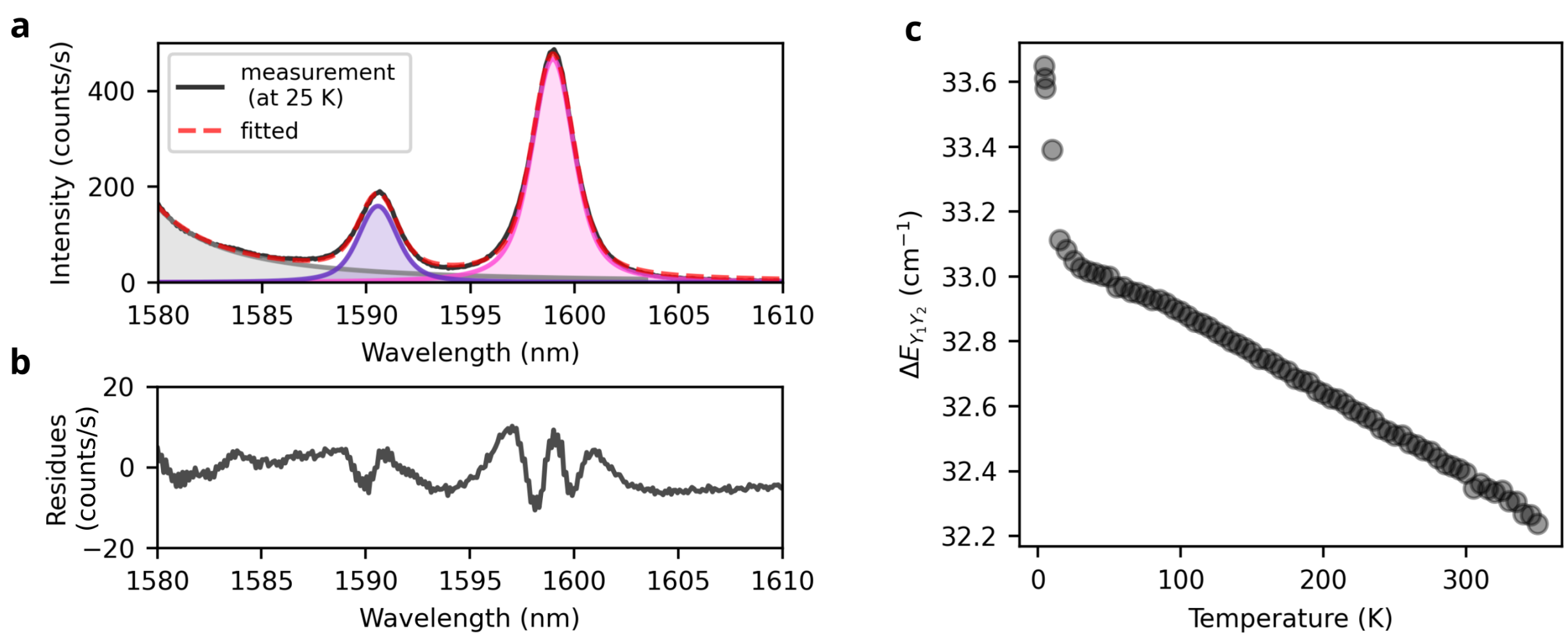

Figure S3: **a**: Partial luminescence spectrum of the transition ${}^4I_{13/2} \rightarrow {}^4I_{15/2}$ in Y_2O_3: Yb^{3+}/Er^{3+} systems showing the $Y_1 \rightarrow Z_6$ and $Y_2 \rightarrow Z_6$ Stark lines and their respective fitted Voigt curves. **b**: Fit residue from the experimental curves. **c**: Energy difference between Y_1 and Y_2 states as a function of the temperature.

Keeping the excitation power at reasonable values is essential to avoid effects such as self-heating, excited state absorption, and stimulated emission, which could jeopardize accurate absolute temperature measurements. We measured the luminescence intensity of the corresponding $Y_1 \rightarrow Z_6$ and $Y_2 \rightarrow Z_6$ lines as a function of the excitation power at room temperature (Fig. S4**a**). We observe that the integrated intensities I follow a power-law $I \propto P^n$, where P is the excitation

irradiance (excitation power divided by the excitation area). As expected for the $^4I_{13/2} \rightarrow {}^4I_{15/2}$ transition below the saturation regime, we obtained $n = 1$.

To experimentally verify Boltzmann assumption (Eq. (1) of the main text), Fig. S4**b** shows R_{Stark} as a function of the internal cryostat temperature, measured with its internal thermometric probe. The calculated ΔE_{eff} from the fitting[S3] agrees with $\Delta E_{Y_1Y_2}$ for temperatures above 15 K. For temperatures below this point, we observe a plateau in the $\ln(R_{\text{Stark}})$ *vs.* T^{-1} curve, where Boltzmann distribution is not attained. This is most probably due to the competition between radiative and non-radiative decay rates, as discussed in ref. [S3]. Therefore, a Boltzmann thermometer using this pair of levels would not be appropriate for $T < 15K$.

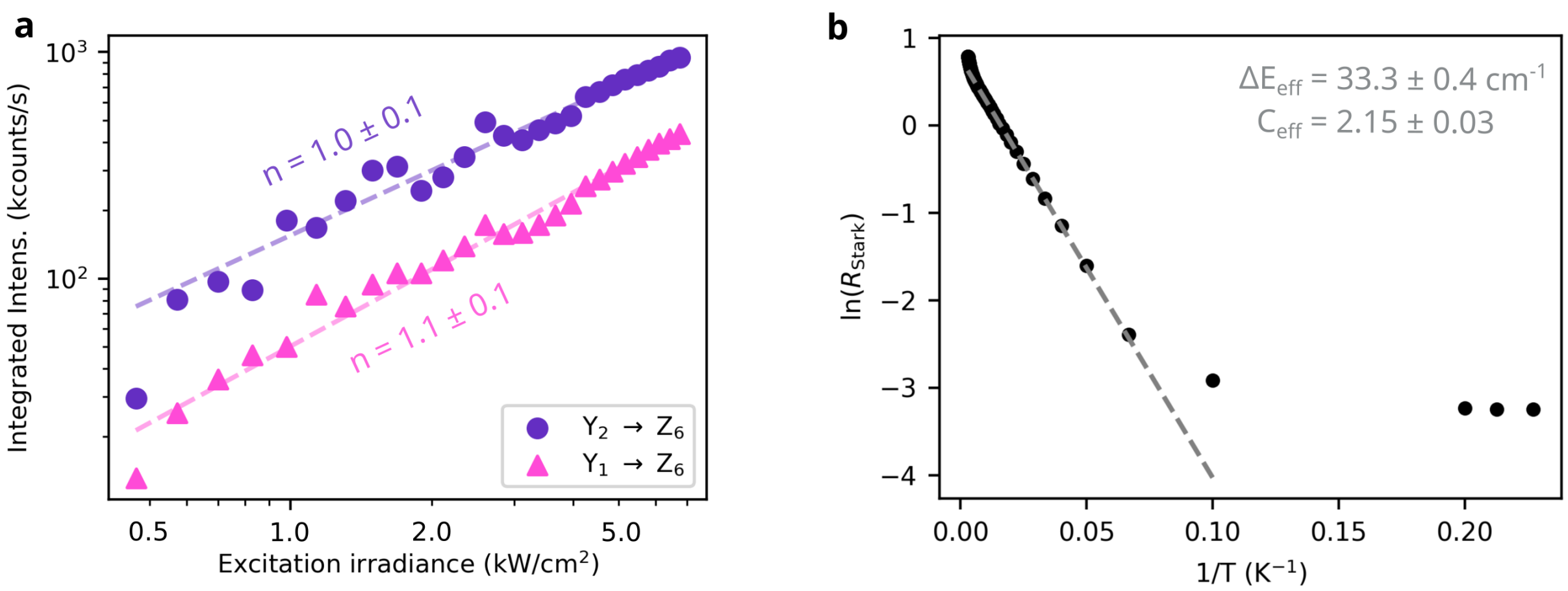

Figure S4: **a**: Intensity of the Stark lines as a function of the excitation irradiance. The dashed curves represent the fittings $I = A \cdot P^n$, with A and n as fitting parameters. The resulting n values are shown within the plot. **b**: Experimental verification of Boltzmann assumption. The dashed line represents a linear fitting for tempertures higher than 15 K, from which we extract C_{eff} and ΔE_{eff} (inset).

S4 Characterization of the Stark lines $E_1 \rightarrow (Z_7 + Z_8)$ and $E_2 \rightarrow (Z_1 + Z_2)$

Two-photon upconversion processes with excitation at 980 nm can populate the $^2H_{11/2}$ and $^4S_{3/2}$ spin-orbit manifolds of the Er^{3+} ions (Fig. S5**a**). These manifolds decay radiatively to the ground state ($^4I_{15/2}$), resulting in luminescence bands in the green spectral region. Fig. S5**b** shows the transitions $^2H_{11/2} \rightarrow {}^4I_{15/2}$ and $^4S_{3/2} \rightarrow {}^4I_{15/2}$ for temperatures between 4.4 and 350.0 K. As

discussed in the main text, we have selected the Stark lines $E_1 \rightarrow (Z_7 + Z_8)$ and $E_2 \rightarrow (Z_1 + Z_2)$ of the $^4S_{3/2}$ band (highlighted in Fig. S5**c**) as thermally coupled pairs for performing absolute primary thermometry.

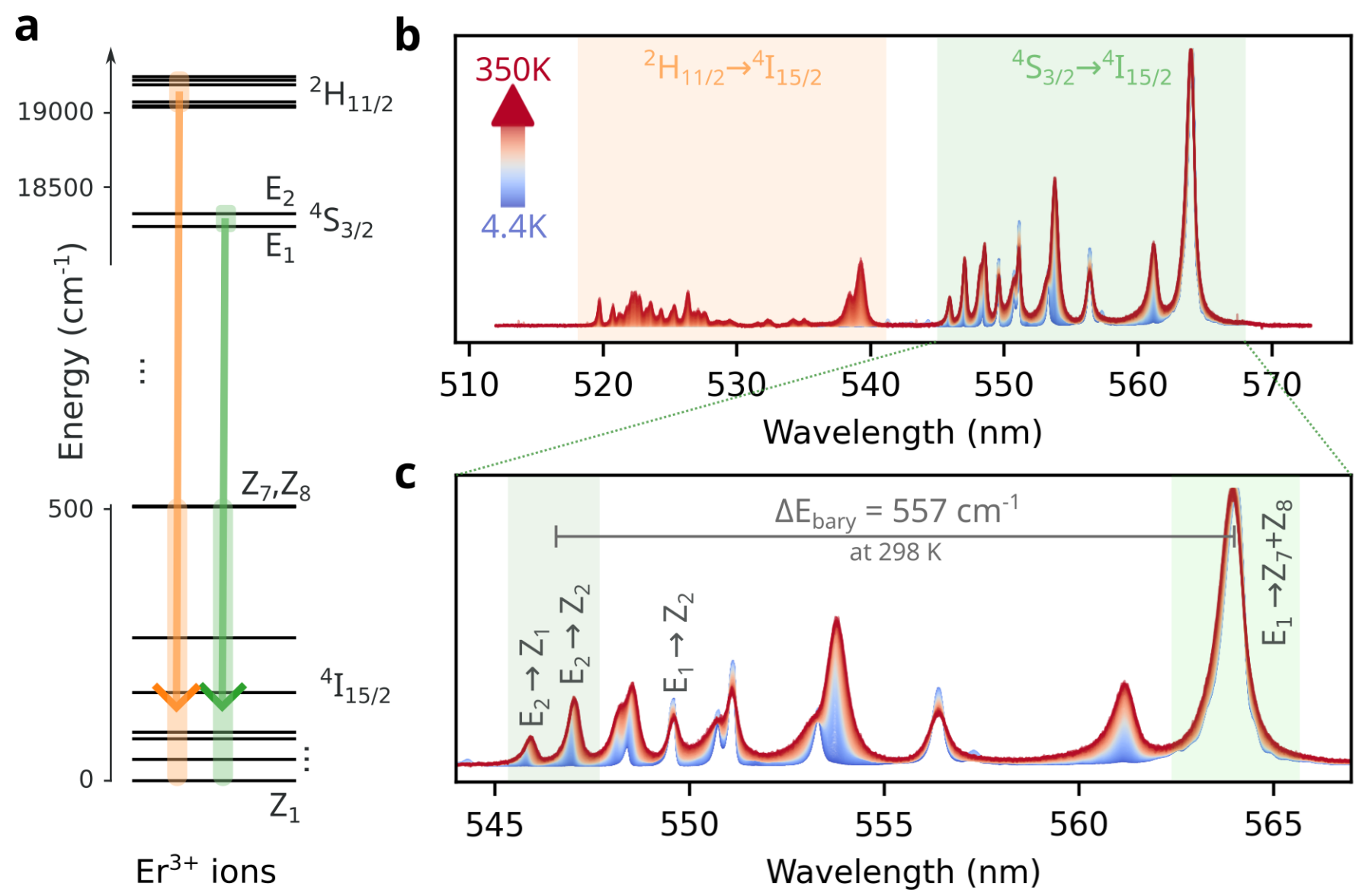

Figure S5: **a**: Simplified energy-level diagram of Er^{3+} ions showing the $^2H_{11/2}$ and $^4S_{3/2}$ manifolds, which are electronically populated after a two-photon excitation at 980 nm. **b**: Luminescence spectra of the transitions $^2H_{11/2} \rightarrow {}^4I_{15/2}$ and $^4S_{3/2} \rightarrow {}^4I_{15/2}$ as a function of the internal temperature of the cryostat. **c**: The same plot as in **b**, but highlighting the Stark lines that will be used for absolute primary optical thermometry.

The selected Stark lines are depicted in Fig. S6**a** and were fitted similarly as in Section S3. The fit residues are shown in Fig. S6**b**. Since $E_1 \rightarrow (Z_7 + Z_8)$ and $E_2 \rightarrow (Z_1 + Z_2)$ lines do not share the same final state, ΔE_{bary} is different from $\Delta E_{E_1E_2}$. To properly measure $\Delta E_{E_1E_2}$ we chose the pair of Stark lines $E_1 \rightarrow Z_2$ and $E_2 \rightarrow Z_2$, as shown in Fig S5**c**. $\Delta E_{E_1E_2}$ is depicted in Fig. S6**c** for temperatures between 4.4 K and 350.0 K, for characterization.

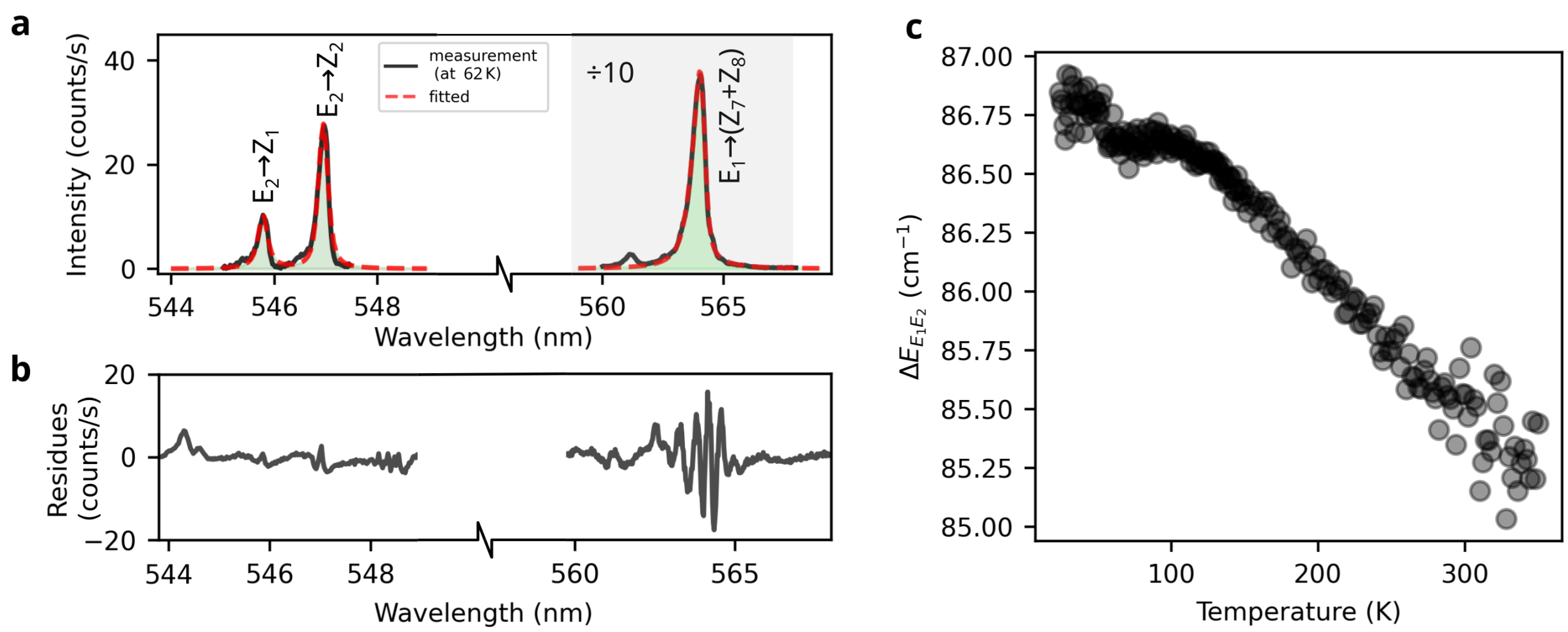

Figure S6: **a**: Partial luminescence spectrum of the transition $^4S_{3/2} \rightarrow {}^4I_{15/2}$ in Y_2O_3: Yb^{3+}/Er^{3+} systems showing the $E_1 \rightarrow (Z_7 + Z_8)$ and $E_2 \rightarrow (Z_1 + Z_2)$ Stark lines and their respective fitted Voigt curves (see main text for details). **b**: Fit residues from the experimental curves. **c**: Energy difference between E_1 and E_2 as a function of the temperature.

Power-law analysis, similarly to that of section S3, reveals $n = 2$ for both transitions (Fig. S7**a**), indicating a non-saturated upconverting process. Similarly, the plot $\ln(R_{\text{Stark}})$ *vs.* T^{-1} curve (Fig. S7**b**), which serve as a verification of the Boltzmann assumption, shows $\Delta E_{\text{eff}} = 83.8 \pm 0.4$ cm^{-1}, in excellent agreement with $\Delta E_{E_1E_2}$ measured through the luminescence spectrum.

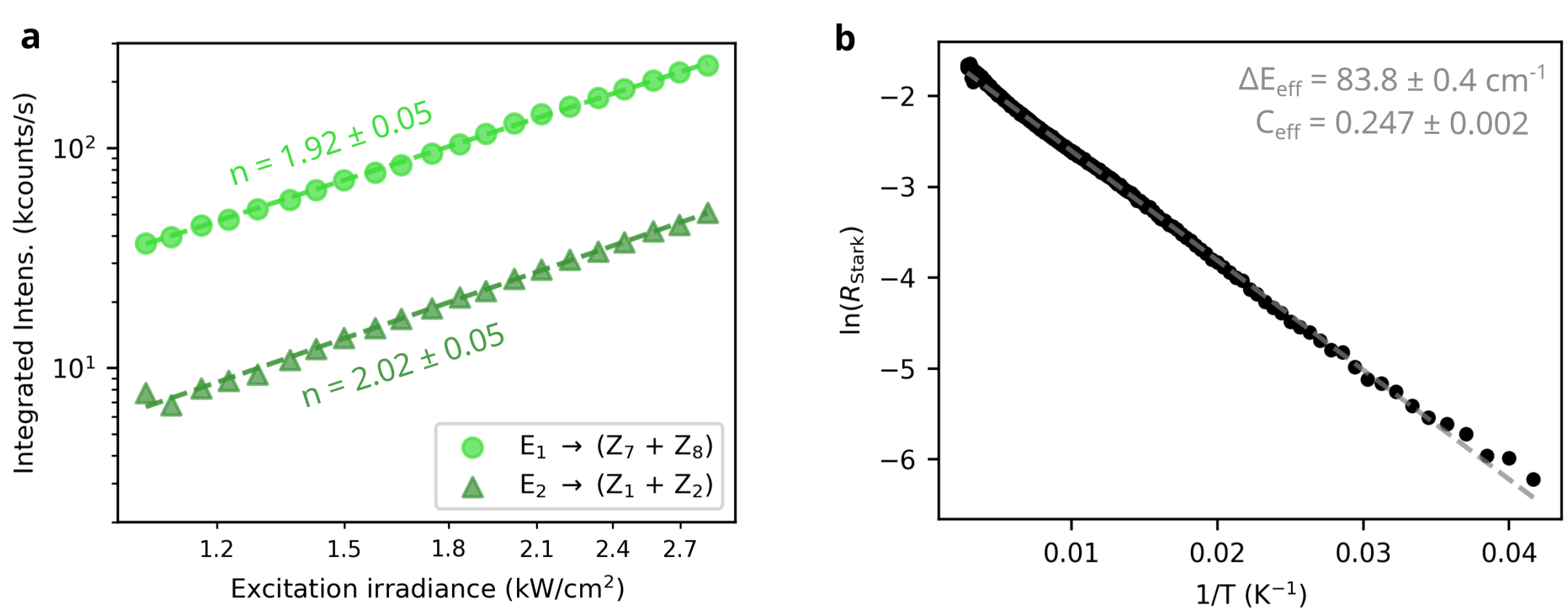

Figure S7: **a**: Intensity of the Stark lines as a function of the excitation irradiance. The dashed lines represent the fittings $I = A \cdot P^n$, with A and n as fitting parameters. **b**: Experimental verification of Boltzmann assumption. The dashed line represents a linear fitting, from which we extract C_{eff} and ΔE_{eff} (inset).

S5 Accuracy and precision

In both proposed methods, the temperature uncertainty is obtained through standard error propagation from the uncertainties of ΔE and C. As discussed in the main text, ΔE is measured directly from the luminescence spectrum, with uncertainties on the order of 0.5 cm^{-1} or less (depending on the resolution of the spectrometer), while C depends on the specific method employed. It is important to note that the thermal uncertainty reflects the thermometer's precision, whereas accuracy corresponds to the deviation between the measured and the actual temperature. Obtaining accuracy requires comparison of the proposed thermometric system with previously calibrated probes.

For absolute primary thermometry based on the measurement of the slope $\mathrm{d}(P \cdot \ln R_{\mathrm{Stark}})/\mathrm{d}P$ in the high-temperature limit (Eq. (3) of the main manuscript), the uncertainty in $\ln C$ is equal to the uncertainty resulting from the linear fitting to obtain the slope. Naturally, this uncertainty calculation should inherit the individual uncertainties of the data point σ_{dp}, as $\sigma_{dp}^2 = (P \cdot \frac{1}{R_{\mathrm{Stark}}} \sigma_R)^2 + (R_{\mathrm{Stark}} \cdot \sigma_P)^2$, where σ_R is the uncertainty of R_{Stark} and σ_P is the uncertainty of P. The errors on the power measurements, σ_P, depend on the current and voltage sensors used. In our case we considered as $\sigma_P = 0.001$ W as given by the equipment.

To compute σ_R, a standard approach is to record multiple spectra at a fixed point and measure the mean and standard deviation of R_{Stark}. Alternatively, as adopted in this work, the uncertainty can be estimated from the Voigt profile fittings of the spectrum. Given that $\int_{-\infty}^{\infty} V(x), \mathrm{d}x = V_0$, the uncertainty in the Stark line areas can be taken as the uncertainty of the fitted parameter V_0, as provided by the fitting routine. This estimate inherently accounts for the residuals of the experimental data and potential correlations among fitting parameters. It should be noted that smaller V_0 values are associated with larger relative uncertainties due to the shot-noise limit.

For absolute thermometry based on the measurement of R_{Stark} at the inflection point, the uncertainty in C was considered equal to that of $R_{\mathrm{Stark}}(P_{\mathrm{infl}})$ multiplied by e^2, i.e., $\sigma_C = \sigma_R \cdot \mathrm{e}^2$ (see main text). The specific error treatment on σ_R depends on the method used to identify the inflection point. When using a Savitzky–Golay filter, the uncertainty depends on the local fitting residuals, and naturally the experimental σ_R must be incorporated in the local fit. Then, P_{infl} is identified with some uncertainty, and the corresponding uncertainty in $R_{\mathrm{Stark}}(P_{\mathrm{infl}})$ is calculated through standard error propagation.

Regardless of the method used to obtain ΔE and C, the temperature measurement uncertainty is given by[S3]

$$\sigma_T^2(T) = \frac{1}{S_r(T)^2}\left[\left(\frac{\sigma_{\Delta E}}{k_\mathrm{B}T}\right)^2 + \left(\frac{\sigma_C}{C}\right)^2 + \left(\frac{\sigma_R}{R}\right)^2\right]. \tag{S5}$$

where we disregarded the correlation between the variables because ΔE and C are measured independently in the absolute primary thermometry approach.

References

(S1) Lienhard, J. H., V; Lienhard, J. H., IV *A Heat Transfer Textbook*, 6th ed.; Phlogiston Press: Cambridge, MA, 2024; Version 6.00.

(S2) Possmayer, T.; Pessoa, A. R.; Galindo, J. A. O.; dos Santos, L. F.; Gonçalves, R. R.; Amaral, A. M.; Menezes, L. de S. Boltzmann Thermometry at Cryogenic Temperatures Exploiting Stark Sublevels in Er^{3+}/Yb^{3+}-Codoped Yttrium Oxide Nanoparticles. *ACS Appl. Mater. Interfaces* **2026**, *18*, 7249–7257.

(S3) Pessoa, A. R.; Menezes, L. de S.; Amaral, A. M. Addressing Discrepancies between Theory and Experiments in Boltzmann Luminescence Thermometry with Ln^{3+} Ions. *J. Phys. Chem. C* **2025**, *129*, 11143–11154.
